# miR-200c suppresses endometriosis by targeting MALAT1 in vitro and in vivo

**DOI:** 10.1186/s13287-017-0706-z

**Published:** 2017-11-07

**Authors:** Zongwen Liang, Yijie Chen, Yuan Zhao, Chaoyi Xu, Anqi Zhang, Qiong Zhang, Danhan Wang, Jing He, Wenfeng Hua, Ping Duan

**Affiliations:** 1 Department of Obstetrics and Gynecology, The Second Affliated Hospital and Yuying Children’s Hospital, Wenzhou Medical University, Wenzhou, 325027 Zhejiang China; 2 Department of Pediatric Surgery, Guangzhou Institute of Pediatrics, Guangzhou Women and Children’s Medical Center, Guangzhou Medical University, Guangzhou, 510623 Guangdong China; 3 Department of Laboratory Medicine and Central Laboratories, Guangdong Second Provincial General Hospital, Southern Medical University, Guangzhou, 510317 Guangdong China

**Keywords:** Endometriosis, Epithelial-to-mesenchymal transition, Competing endogenous RNA, Nanoparticle, miR-200c, MALAT1

## Abstract

**Background:**

Endometriosis is a common, benign, and estrogen-dependent disease characterized by pelvic pain and infertility. To date, the pathogenesis of endometriosis remains unclear. Recent studies have demonstrated that noncoding RNAs, including microRNAs and long noncoding RNAs, play important roles in the development of endometriosis.

**Methods:**

Expression profiling of miRNAs in endometrial tissue was characterized using microarrays. The most differentially expressed miRNAs were confirmed using quantitative reverse transcriptase-polymerase chain reaction analysis in additional ectopic endometrial (*n* = 27) and normal endometrial (*n* = 12) tissues. For in-vitro functional studies, 5-ethynyl-2′-deoxyuridine incorporation assay, Transwell assay, and dual-luciferase reporter assay were used to measure the proliferation, migration, and luciferase activity of miR-200c and the predicted targets of miR-200c in primary endometrial stromal cells (HESCs) derived from human endometrial biopsies, respectively. For in-vivo therapeutic interventions, polymeric nanoparticles of polyethylenimine–polyethylene glycol–arginine–glycine–aspartic acid were used for delivery of miR-200c mimic and inhibitor to determine the therapeutic effect of miR-200c in a rat model of endometriosis.

**Results:**

Exogenous overexpression of miR-200c inhibited the proliferation and migration of HESCs, which were mainly regulated by metastasis-associated lung adenocarcinoma transcript 1 (MALAT1). In contrast, inhibition of miR-200c promoted the proliferation and migration of HESCs, while the simultaneous silencing of MALAT1 expression exerted the opposite effects. We demonstrated that expression of MALAT1 in ectopic endometrial specimens was negatively correlated with that of miR-200c and that MALAT1 knockdown increased the level of miR-200c in HESCs. Moreover, the transfection of endometrial stromal cells with the miR-200c mimic or MALAT1 siRNAs decreased the protein levels of mesenchymal markers ZEB1, ZEB2, and N-cadherin and increased the protein levels of the epithelial marker E-cadherin. Furthermore, using a rat endometriosis model, we showed that local delivery of the miR-200c mimic significantly inhibited the growth of ectopic endometriotic lesions.

**Conclusions:**

The MALAT1/miR-200c sponge may be a potential therapeutic target for endometriosis.

**Electronic supplementary material:**

The online version of this article (doi:10.1186/s13287-017-0706-z) contains supplementary material, which is available to authorized users.

## Background

Endometriosis is characterized by the presence and growth of endometrial glands and stroma outside the uterine cavity. The disease affects 6–10% of reproductive-age women. Endometriosis has multiple manifestations, such as dysmenorrhea, pelvic pain, pelvic mass, infertility, and cancerous lesions, and it can severely affect the quality of life of patients. However, the pathogenesis of endometriosis is still unclear. The current gold standard for the diagnosis of endometriosis is surgical assessment by laparoscopy, and there are no reliable noninvasive diagnostic markers for endometriosis [1–3]. Thus, it is urgent to explore the mechanism of endometriosis and find a noninvasive biomarker for the diagnosis and treatment of this disease.

MicroRNAs (miRNAs) are a class of small, noncoding, single-stranded RNAs approximately 20–24 nucleotides in length, which regulate gene expression at the post-transcriptional level by interacting with the 3′-untranslated regions of mRNAs and triggering either translational repression or RNA degradation. miRNAs participate in various cellular processes, such as cell proliferation, apoptosis, invasion, and migration [4, 5]. Long noncoding RNAs (lncRNAs) are another class of noncoding RNAs (ncRNAs). lncRNAs are transcripts that are longer than 200 nucleotides with extremely limited protein-coding potential. lncRNAs regulate multiple processes, including proliferation, apoptosis, cell differentiation, genomic imprinting, RNA alternative splice, and chromatin modification [6, 7]. Additionally, lncRNAs are involved in the pathogenesis and progression of many human diseases, including endometriosis [8–10]. Wang et al. [8] reported that lncRNA AC002454.1 can participate in the pathogenesis of endometriosis by regulating CDK6 to disrupt the cell cycle. Expression of lncRNA SRA has been found to be associated with expression of estrogen receptor alpha and proliferation of endometriotic stromal cells [11]. LncRNA H19 expression has recently been shown to decrease in eutopic endometrial tissues, and the restoration of H19 expression in endometriotic stromal cells can promote cell proliferation [12]. However, the function of lncRNAs in endometriosis remains unknown, and related studies are still scarce.

Epithelial-to-mesenchymal transition (EMT) is the process by which epithelial cells acquire mesenchymal phenotypes, thereby becoming highly motile. These changes are thought to be prerequisites for the formation of endometriotic lesions [13]. During EMT, expression of the epithelial marker E-cadherin is decreased, while expression of mesenchymal markers, such as N-cadherin and vimentin, is increased [14]. ZEB1 and ZEB2 mRNAs are well-described targets of the miR-200 family, and many reports have suggested that miR-200 family members block EMT by inhibiting ZEB1 and ZEB2 expression [15].

In the current study, we found that miR-200c suppresses the proliferation and migration of endometrial stromal cells by downregulating metastasis-associated lung adenocarcinoma transcript 1 (MALAT1), a lncRNA which in turn functions as a miR-200c decoy and abolishes the suppressive effect of miR-200c. Mechanistically, the MALAT1/miR-200c sponge acts, at least in part, by regulating expression of ZEB1 and ZEB2 in endometrial stromal cells. In addition, we performed therapeutic interventions with the miR-200c mimic and inhibitor in a rat model of endometriosis, and our results demonstrated the potential therapeutic effect of miR-200c in endometriotic lesions. Overall, these findings suggest that the MALAT1/miR-200c sponge may represent a potential and novel therapeutic target for endometriosis.

## Methods

### Clinical specimens

Ectopic endometrial tissues were collected from endometriosis patients, and normal endometrial tissues from uterine leiomyoma or hysterectomy in patients with grade II–III cervical intraepithelial neoplasia (CIN). All of the tissue samples were collected at the time of surgery at the Second Affiliated Hospital of Wenzhou Medical University. Informed consent was obtained from each patient, and the study was approved by the Institutional Ethics Review Board of the Second Affiliated Hospital of Wenzhou Medical University. The samples were placed in liquid nitrogen immediately after collection and subsequently stored in liquid nitrogen. All of the patients had normal ovulation with regular menstrual cycles, and none of the patients had received steroid hormonal medications within a minimum of 3 months.

### Isolation of primary endometrial stromal cells and cell culture

Primary endometrial stromal cells (HESCs) were isolated according to a method described previously [16]. Briefly, ectopic endometrial tissues were minced and digested in Hank’s balanced salt solution containing HEPES (25 mmol/ml), 1% penicillin/streptomycin, collagenase (1 mg/ml, 15 U/mg), and deoxyribonuclease (0.1 mg/ml, 1500 U/mg) at 37 °C for 60 minutes in a shaking water bath. The dispersed endometrial cells were separated by filtration through a 40-μm cell strainer (Falcon, New York, USA). The endometrial stromal cells were plated in 75-cm^2^ Falcon tissue culture flasks (BD Biosciences, MA, USA) and maintained in Ham’s F12/DMEM (1:1) supplemented with heat-inactivated 10% fetal bovine serum (FBS; Gibco BRL, Gaithersburg, MD, USA) and antibiotics (100 IU/ml penicillin, 100 μg/ml streptomycin, and 0.25 μg/ml amphotericin B) at 37 °C in a humidified atmosphere containing 5% CO_2_. The purities of cells were checked by immunohistochemical staining for vimentin (DAKO Corp., Tokyo, Japan). Cultured endometrial stromal cells were used between passages 3 and 5.

### RNA extraction and quantitative real-time PCR

Total RNA was extracted from frozen tissues or primary cultured cells using TRIzol reagent (Invitrogen, Carlsbad, USA). First-strand cDNA was synthesized from 1 μg of total RNA using a Reverse Transcription Kit (Toyobo Co., Osaka, Japan) and was then used for quantitative real-time PCR (qPCR) analysis. For the analysis of miRNA expression, RNA was reverse transcribed using Bulge-Loop™ miRNA-specific RT primers (RiboBio, Guangzhou, China). The qPCR protocol was carried out using SYBR Green PCR master mix (Bio-Rad, Hercules, CA, USA) and an Applied Biosystems 7300 Real-Time PCR System. The PCR primer pairs specific for MALAT1 were 5′-GAATTGCGTCATTTAAAGCCTAGTT-3′ (sense) and 5′-GTTTCATCCTACCACTCCCAATTAAT-3′ (antisense), and the primers specific for XIST were 5′-ACATGCCTGGCACTCTAGCA-3′ (sense) and 5′-AAACATGGAAATGGGTAAGACACA-3′ (antisense). The small nuclear RNA (snRNA) U6 or GAPDH was used as an internal control to normalize the expression levels of the different genes. The PCR primers specific for GAPDH were 5′-AACGGATTTGGTCGTATTGG-3′ (sense) and 5′-CTTCCCGTTCTCAGCCTTG-3′ (antisense), and the primers specific for U6 snRNA were 5′-CTCGCTTCGGCAGCACA-3′ (sense) and 5′-AACGCTTCACGAATTTGCGT-3′ (antisense).

### Western blot analysis

After a 48-hour transfection, HESCs were lysed using RIPA buffer (100 mM Tris–HCl, 300 mM NaCl, 2% NP40, and 0.5% sodium deoxycholate) supplemented with Protease Inhibitor Cocktail (Roche Molecular Biochemicals). Protein concentrations were assayed using a BCA Protein Assay Kit (Pierce, Rockford, IL, USA). Identical quantities of proteins were separated via SDS-PAGE and transferred to polyvinylidene difluoride membranes (Millipore Corporation, Bedford, MA, ISA). The membranes were incubated overnight at 4 °C with the indicated primary antibodies against the following: ZEB1 (#3396, 1:1000 dilution; Cell Signaling), ZEB2 (HPA003456, 1:100 dilution; Sigma-Aldrich, St. Louis MO, USA), N-cadherin (#13116, 1:1000 dilution; Cell Signaling), E-cadherin (#3195, 1:1000 dilution; Cell Signaling), and α-Tubulin (T6199, 1:2000 dilution; Sigma-Aldrich). Goat anti-mouse (A0412) and goat anti-rabbit (A6154) peroxidase-conjugated secondary antibodies were obtained from Sigma-Aldrich. Protein bands were visualized using a chemiluminescence kit (Pierce). α-Tubulin was used as a loading control for the western blot analyses.

### Cell transfection

The miR-200c mimic, miR-200c inhibitor, siRNA against MALAT1, and their respective negative controls (scrambled oligos) were obtained from RiboBio. miRNA and siRNA transfections were performed using Lipofectamine 2000 (Life Technologies, Gaithersburg, MD, USA) according to the manufacturer’s instructions. The target sequences of siRNAs were as follows: siMALAT1-1, 5′-GATCCATAATCGGTTTCAA-3′; and siMALAT1-2, 5′-CACAGGGAAAGCGAGTGGTTGGTAA-3′.

### Cell proliferation assay

Cell proliferation was measured using EdU incorporation assays. Cells were starved in serum-free medium for 24 hours before the EdU incorporation assay. After a 48-hour transfection, cell proliferation was determined with an EdU Assay Kit (RiboBio) according to the manufacturer’s instructions. All experiments were repeated three times.

### Cell migration assay

Cell migration assay was conducted in 24‐well Transwell chambers (Corning Costar Corporation, Corning, NY, USA). After 24-hour transfection, cells were suspended in 0.5 ml serum-free medium and loaded on the top chamber, and medium supplemented with 10% FBS was added to the bottom chamber as the chemoattractant. After 36 hours of incubation at 37 °C, cells that had migrated through the membrane were fixed with formaldehyde and stained with 1% crystal violet for 30 minutes. The migrated cells attached to the lower surface of the membrane insert were counted in five random fields under a 20× objective lens and imaged using SPOT imaging software (Nikon, Tokyo, Japan). Three independent experiments were performed.

### Dual-luciferase reporter assay

For luciferase reporter assays, the two putative miR-200c binding sites on MALAT1 RNA were cloned downstream of the cytomegalovirus (CMV) promoter in a pMIR-REPORT vector (Ambion, Carlsbad, CA, USA). The miR-200c binding sites at positions 3483 and 5466 containing partial MALAT1 sequences are located at 3410–3809 nt and 5205–5634 nt, respectively. Luciferase activity was measured using a Dual-Luciferase Reporter Assay System (Promega, Madison, WI, USA) according to the manufacturer’s instructions. Briefly, pMIR-REPORT-MALAT1 or pMIR-REPORT-MALAT1-mut was cotransfected with the miR-200c mimic, the miR-200c inhibitor, or the corresponding negative controls into HESCs using Lipofectamine 2000 according to the manufacturer’s instructions. Luciferase activity was normalized to Renilla luciferase activity at 48 hours after transfection.

### Synthesis of the PEI–PEG–RGD and polyelectrolyte complexes

Branched polyethylenimine (PEI, 25 kDa) and arginine–glycine–aspartic acid (RGD) peptides were purchased from Sigma-Aldrich. *N*-hydroxysuccinimide–polyethylene glycol–maleimide (NHA–PEG–Mal, 3.5 kDa) was obtained from Jenkem Technology Company (Beijing, China). The 10-kDa cutoff centrifugal filters were purchased from Millipore.

The PEI–PEG polymer complex was synthesized as described previously [17]. Briefly, NHS–PEG–Mal (8 mg/ml) was added to PEI (4 mg/ml) in NaOH solution (pH 8.2) and placed in a 37 °C incubator for 1 hour. The resultant PEI–PEG complexes were purified using a 10-kDa cutoff filter to remove the free NHS–PEG–Mal. The PEI–PEG complexes were then washed three times with deionized water and resuspended in water (pH 7.2). After purification of the PEI–PEG complexes, they were incubated with excess RGD peptides (dissolved in pH 7.2 water, 1 mg/ml), in which the cysteine group was covalently reacted with maleimide, at 37 °C for 12 hours to form PEI–PEG–RGD conjugates. Finally, the obtained PEI–PEG–RGD conjugates were purified via dialysis (molecular weight cutoff 8–14 kDa) against deionized water to eliminate the unbound RGD. In the current study, we used the PEI–PEG–RGD conjugates at 1 mg/ml (applied as PEI concentration) for nucleic acid delivery. Aiming to optimize the nitrogen to phosphate (N/P) ratio, miRNA contents were predetermined and mixed with various amounts of PEI–PEG–RGD. The miRNA@PEI–PEG–RGD formulation was spontaneously achieved due to electrostatic interaction between cationic PEI and the negative charge on miRNAs after 30 minutes of mixing at room temperature. After that, the samples were subjected to agarose gel electrophoresis to determine the optimal N/P ratio when all the miRNAs were trapped by PEI–PEG–RGD. To evaluate the transfection efficiency and cytotoxicity of PEI–PEG–RGD, see Additional file 1.

### Establishment of the endometriosis rat model

Six-week-old female nonpregnant Sprague–Dawley rats (180–200 g) were obtained from the Laboratory Animal Research Center of Wenzhou Medical University. All animal handling and experimental procedures were approved by the Animal Experimental Ethics Committee of Wenzhou Medical University. The rat model of endometriosis was established by the autotransplantation of endometrium as described previously with minor modifications [18]. The rats were anesthetized with 4% chloral hydrate (4 g/100 ml), and a small incision was made at the center of the abdomen. The left uterine horn was excised and immediately placed in saline solution, and the endometrium was carefully separated from muscles. The endometrium was cut into two fragments approximately 5 mm on each side. A subcutaneous pocket was fashioned on each side of the abdominal wall, and the uterine segments were placed in the left and right spaces with the endometrium facing the abdominal muscle. The rats were subcutaneously injected with 30 μg/kg benzoic estradiol immediately after the first laparotomy, and the injection was repeated 10 days later to promote the growth of the autografts. The volume of ectopic endometrial cyst was calculated using the formula *V* = 1/2 (*L* × *W* ^2^).

The rats were divided into six groups, and equal volumes of various solutions were injected into endometriotic lesions. The mimic NC group and the miR-200c mimic group received 1 nmol RNA; the inhibitor NC group and the miR-200c inhibitor group received 2.5 nmol of RNA. All RNA oligos were loaded into PEI–PEG–RGD nanocarriers and labeled with Vivo Tag-S 680 (Perkin Elmer, USA) for in-vivo fluorescence imaging.

### Statistical analysis

The data are presented as the mean ± standard deviation (SD) from at least three independent experiments. Statistical significance was measured using Student’s *t* test (two-tailed). Differences were considered significant at *P* < 0.05.

## Results

### miR-200c is frequently downregulated in endometriosis

**Fig. 1:**
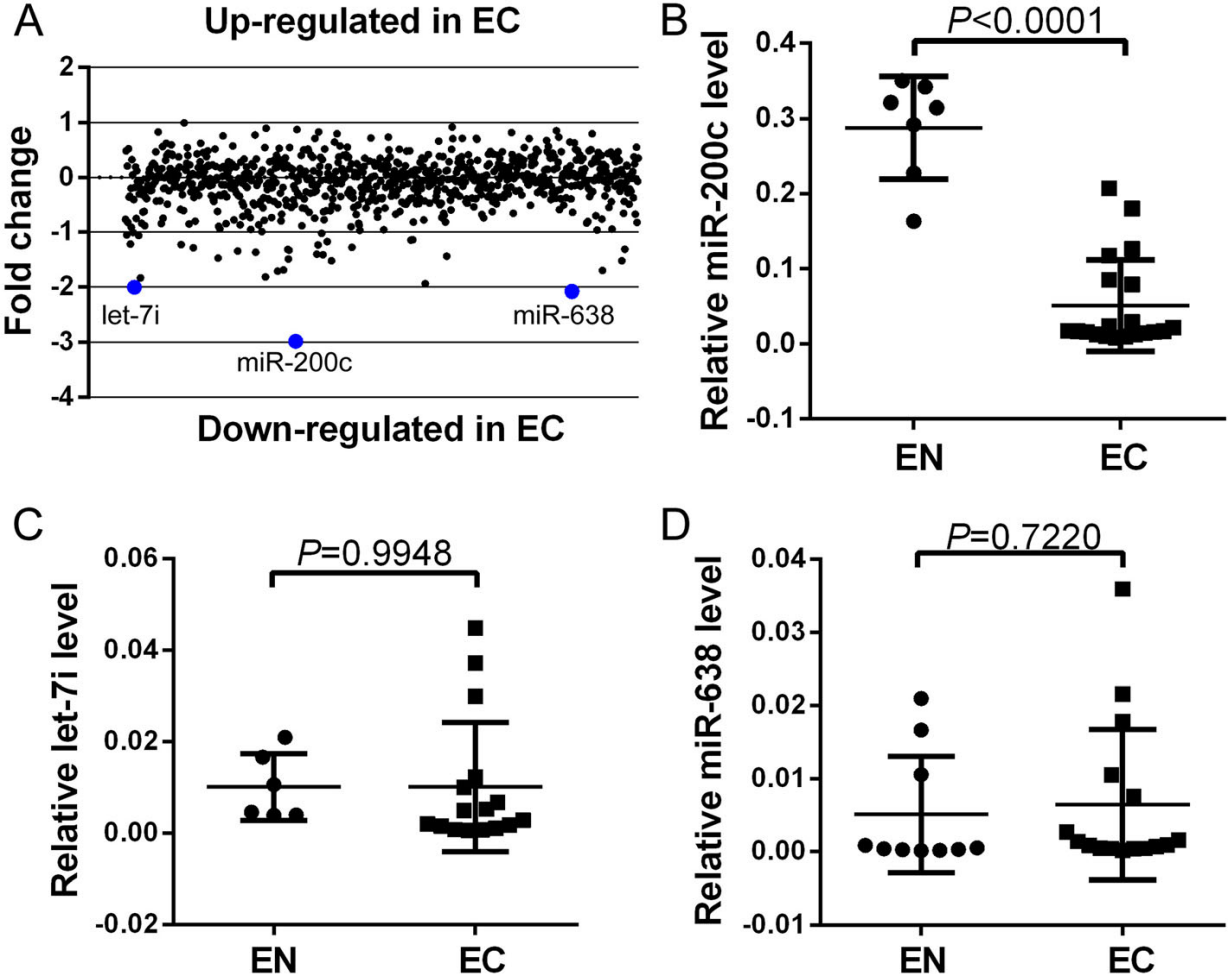
miR-200c expression is downregulated in ectopic endometrial tissues. **a** Expression profiling of miRNAs in EC and EN tissues. **b**–**d** qPCR analysis of miR-200c (**b**), miR-638 (**c**), and let-7i (**d**) expression in 27 EC and 12 EN tissues. Transcript levels normalized according to U6 expression. All data shown as mean ± SD of three independent experiments. EC ectopic endometrial, EN normal endometrial

To identify the differentially expressed miRNAs in endometrial tissues, a miRNA microarray analysis was performed on three ectopic endometrial (EC) and three normal endometrial (EN) tissues. Compared with those in EN tissues, three miRNAs (miR-200c, miR-638, and let-7i) were significantly downregulated in EC tissues (fold change cutoff ≥ 2.0 and *P* < 0.05) (Fig. 1a). Further, we performed qRT-PCR analysis using additional EC and EN tissues to confirm the results of microarray analysis. As shown in Fig. 1b, miR-200c was significantly downregulated in EC tissues (*n* = 27) compared with in EN tissues (*n* = 12) (*P* < 0.0001). However, there were no significant differences in miR-638 and let-7i levels between EC and EN tissues (Fig. 1c, d).

### Restoration of miR-200c expression in endometrial stromal cells suppresses cellular migration and proliferation

**Fig. 2:**
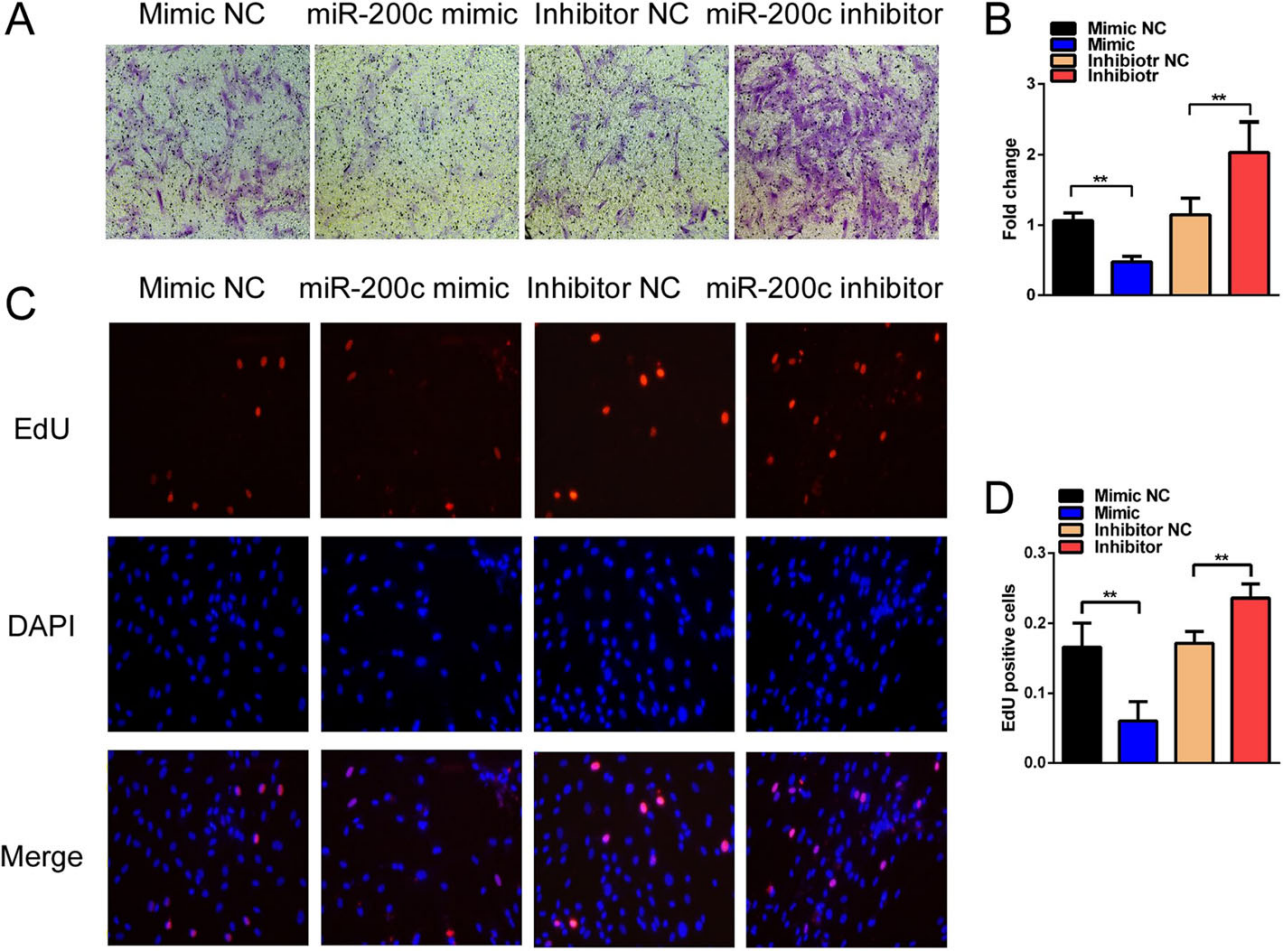
miR-200c suppresses the proliferation and migration of HESCs in vitro. **a** Transwell assay to evaluate the migration of HESCs treated with the miR-200c mimic or inhibitor. Migrated cells were stained with crystal violet (magnification, 200×). **b** Number of migrated cells was calculated and is depicted in the bar chart. **c** EdU assay to evaluate the proliferation of HESCs treated with the miR-200c mimic or inhibitor (magnification, 200×). **d** Number of proliferating cells was calculated and is depicted in the bar chart. All data are shown as the means ± SD of three independent experiments (**P < 0.01, Student’s *t* test). EdU 5-ethynyl-2′-deoxyuridine, Mimic NC negative control miRNA mimic, Inhibitor NC negative control miRNA inhibitor

To better understand the biological function of miR-200c in endometriosis, primary endometrial stromal cells (HESCs) derived from human endometrial biopsies were transfected with the miR-200c mimic or inhibitor to overexpress or suppress miR-200c expression, respectively. As shown in Fig. 2a, b, Transwell assays revealed that miR-200c overexpression suppressed the migration of HESCs, whereas the opposite effect was observed when this miRNA was inhibited. Moreover, the results of a 5-ethynyl-2′-deoxyuridine (EdU) incorporation assay indicated that miR-200c overexpression substantially reduced the rate of cell proliferation. In contrast, the miR-200c inhibitor significantly promoted cell proliferation (Fig. 2c, d). Therefore, miR-200c suppresses the migration and proliferation of endometrial stromal cells in vitro.

### miR-200c directly targets the lncRNA MALAT1

**Fig. 3:**
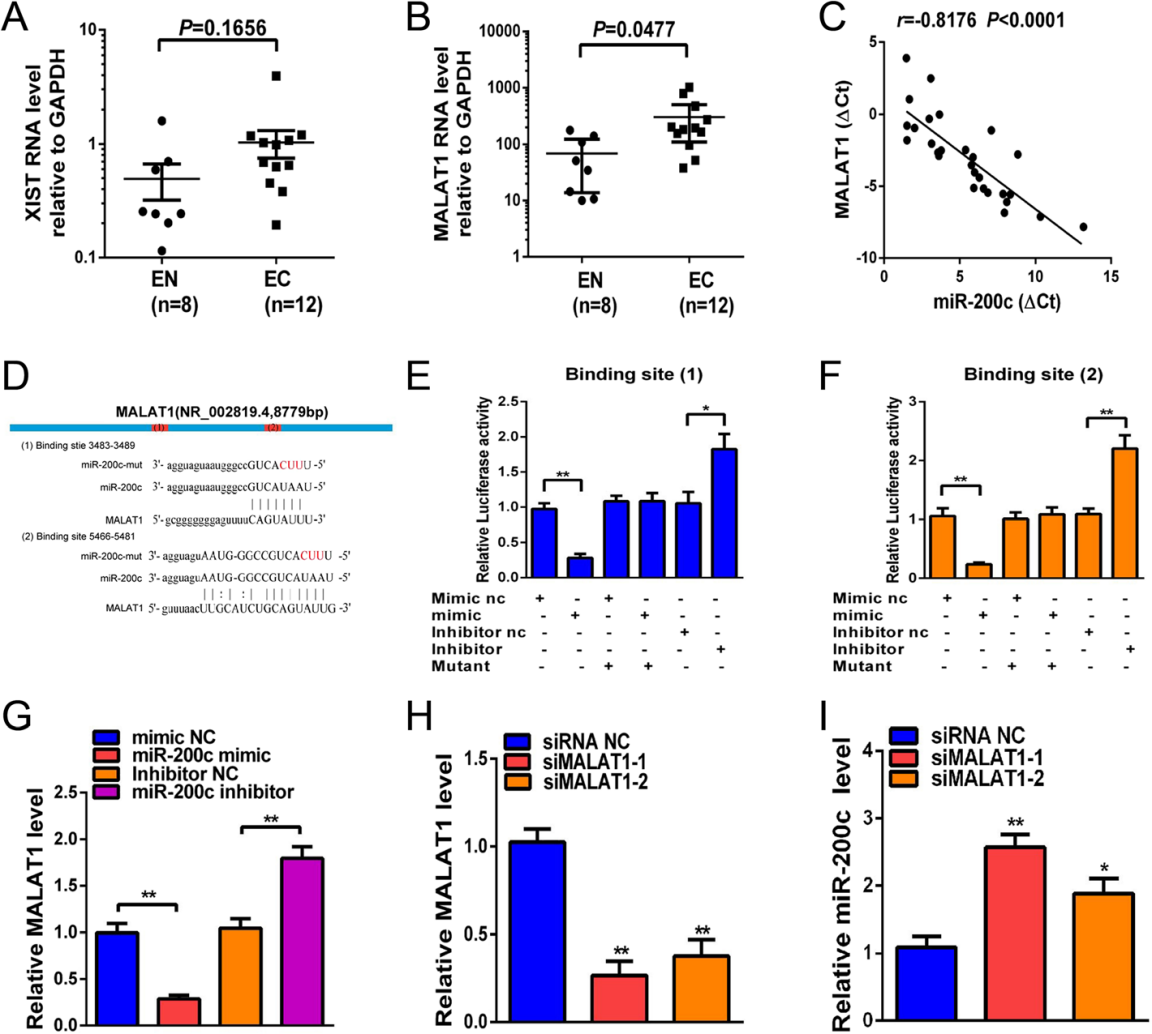
miR-200c reduces the MALAT1 level. **a, b** qRT-PCR showing relative RNA levels of XIST (**a**) and MALAT1 (**b**) in 12 EC and eight EN tissues. **c** Pearson correlation analysis of miR-200c and MALAT1 RNA levels in EC tissues represented by Pearson *R* scores (*n* = 27, *R* < 0 denotes negative correlation). **d** Schematic representation of wild-type and mutant pMIR-REPORT-MALAT1 miRNA expression vectors used in luciferase reporter assays. Three nucleotides altered in the mutant binding site are colored red. **e, f** Relative luciferase activities in HESCs corresponding to binding site 1 (**e**) and binding site 2 (**f**) after cotransfection with the pMIR-REPORT-MALAT1 reporter and the miR-200c mimic (50 nM), mimic NC, miR-200c inhibitor (100 nM), or inhibitor NC for 48 hours. **g** qRT-PCR analysis of MALAT1 expression in HESCs after transfection with the miR-200c mimic (50 nM), mimic NC, miR-200c inhibitor (100 nM), or inhibitor NC for 24 hours. **h, i** Relative RNA levels of MALAT1 (**h**) and miR-200c (**i**) in HESCs that were transiently transfected with siMALAT1(50 nM) or siNC for 24 hours. All data shown as mean ± SD of three independent experiments (*P < 0.05 and **P < 0.01, Student’s *t* test). EC ectopic endometrial, EN normal endometrial, MALAT1 metastasis-associated lung adenocarcinoma transcript 1, Mimic NC negative control miRNA mimic, Inhibitor NC negative control miRNA inhibitor, siRNA small interfering RNA, siRNA NC negative control siRNA

To gain insight into the mechanisms underlying the suppressive function of miR-200c in endometrial stromal cells, we next identified its target genes. Recent studies have revealed that lncRNAs have crucial roles in endometriosis [9]. In addition, many lncRNAs have been reported to function as competing endogenous RNAs (ceRNAs) that can competitively bind to common miRNAs [19]. Based on these findings, we used an miRNA–lncRNA interaction analysis program, starBase v2.0 [20], to screen for potential lncRNAs targeted by miR-200c. Two lncRNAs (MALAT1 and XIST) were identified as possible targets of miR-200c. Next, we used qRT-PCR to determine the MALAT1 and XIST expression in EC and EN tissues. As shown in Fig. 3a, b, expression of XIST was not significantly different between the EC and EN tissues (*P* = 0.1656); however, expression of MALAT1 was significantly upregulated in EC tissues compared with that in EN tissues (*P* = 0.0477). Moreover, the level of MALAT1 was negatively correlated with the miR-200c level (*P* < 0.0001, *r* = −0.8176) (Fig. 3c). Next, we used RNA hybridization programs to demonstrate that the MALAT1 sequence (NR_002819.2) contains two putative binding sites for miR-200c at 3483–3489 bp and 5466–5481 bp (Fig. 3d). To validate the predicted miR-200c binding sites, luciferase reporter assays were performed. miR-200c overexpression resulted in a significant decrease in luciferase activity, whereas the opposite effect was observed when this miRNA was inhibited (Fig. 3e, f). Moreover, mutations in the predicted miR-200c binding sites abolished this effect (Fig. 3d–f). Furthermore, the level of MALAT1 was significantly lower in cells transfected with the miR-200c mimic than in cells transfected with the negative control miRNA mimic (mimic NC) (Fig. 3g). In contrast, the miR-200c inhibitor significantly restored the MALAT1 RNA level in HESCs (Fig. 3g). To further investigate the correlation between miR-200c and MALAT1 expression in endometrial stromal cells, MALAT1 expression was knocked down in HESCs, which resulted in a significant increase in expression of miR-200c (Fig. 3h, i). Therefore, MALAT1 is a target of miR-200c.

### miR-200c suppresses expression of mesenchymal marker genes by downregulating MALAT1

**Fig. 4:**
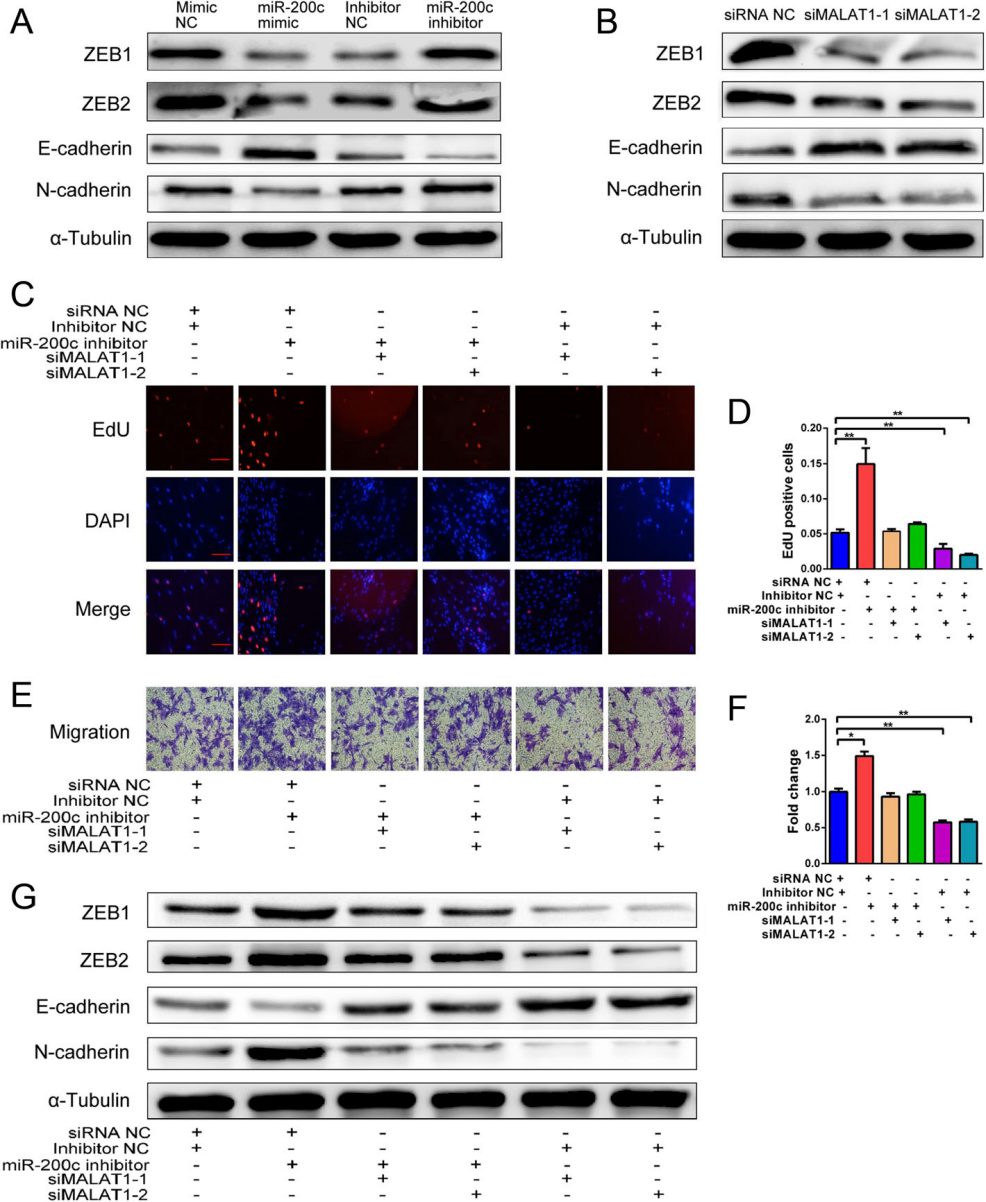
miR-200c regulates EMT in HESCs by downregulating MALAT1. **a** Western blot analysis of ZEB1, ZEB2, N-cadherin, and E-cadherin expression after transfection of HESCs with the miR-200c mimic (50 nM), NC mimic, miR-200c inhibitor (100 nM), or NC inhibitor for 48 hours. **b** Western blot analysis of ZEB1, ZEB2, N-cadherin, and E-cadherin levels after transfection of HESCs with siMALAT1(50 nM) or siNC for 48 hours. **c** EdU assay to evaluate the proliferation of HESCs treated with the miR-200c inhibitor (100 nM), NC inhibitor, siMALAT1 (50 nM), or siNC for 48 hours (magnification, 200×). **d** Number of proliferating cells calculated, depicted in the bar chart. **e** Transwell assay to evaluate the migration of HESCs treated with the miR-200c inhibitor (100 nM), NC inhibitor, siMALAT1 (50 nM), or siNC for 48 hours (magnification, 200×). **f** Number of migrated cells calculated, depicted in the bar chart. **g** Western blot analysis of ZEB1, ZEB2, N-cadherin, and E-cadherin expression after transfection of HESCs with the miR-200c inhibitor (100 nM), NC inhibitor, siMALAT1 (50 nM), or siNC for 48 hours. All data shown as mean ± SD of three independent experiments (*P < 0.05 and **P < 0.01, Student’s *t* test). EdU 5-ethynyl-2′-deoxyuridine, MALAT1 metastasis-associated lung adenocarcinoma transcript 1, Mimic NC negative control miRNA mimic, Inhibitor NC negative control miRNA inhibitor, siRNA small interfering RNA, siRNA NC negative control siRNA

To define the role of miR-200c in the development and progression of endometriosis, we examined its effects on EMT regulation. An increase in miR-200c levels in HESCs by transfection of the miR-200c mimic repressed ZEB1 and ZEB2 protein levels (Fig. 4a). In contrast, the miR-200c inhibitor significantly increased the protein levels of ZEB1 and ZEB2 in HESCs (Fig. 4a). Because ZEB1 and ZEB2 are important transcriptional repressors of E-cadherin expression in EMT regulation, the repression of ZEB1 and ZEB2 in HESCs upregulated E-cadherin and downregulated N-cadherin expression, and vice versa (Fig. 4a). Similarly, western blot analysis showed that the silencing of MALAT1 by siRNAs decreased the protein levels of ZEB1, ZEB2 and N-cadherin and increased the protein level of E-cadherin (Fig. 4b). Moreover, functional studies showed that HESCs transfected with the miR-200c inhibitor demonstrated a significant increase in proliferation and migration; however, silencing of MALAT1 resulted in a significant reduction in proliferation and migration and abolished the effect of the miR-200c inhibitor on HESCs (Fig. 4c–f). Further, western blot analysis confirmed the effect of the miR-200c inhibitor on expression of ZEB1, ZEB2, E-cadherin, and N-cadherin, and these changes in gene expression were reversed by MALAT1 knockdown (Fig. 4g). The suppression of proliferation and migration by miR-200c therefore depends, to a certain extent, on the downregulation of MALAT1.

### miR-200c suppresses of the growth of endometriotic lesions in vivo

**Fig. 5:**
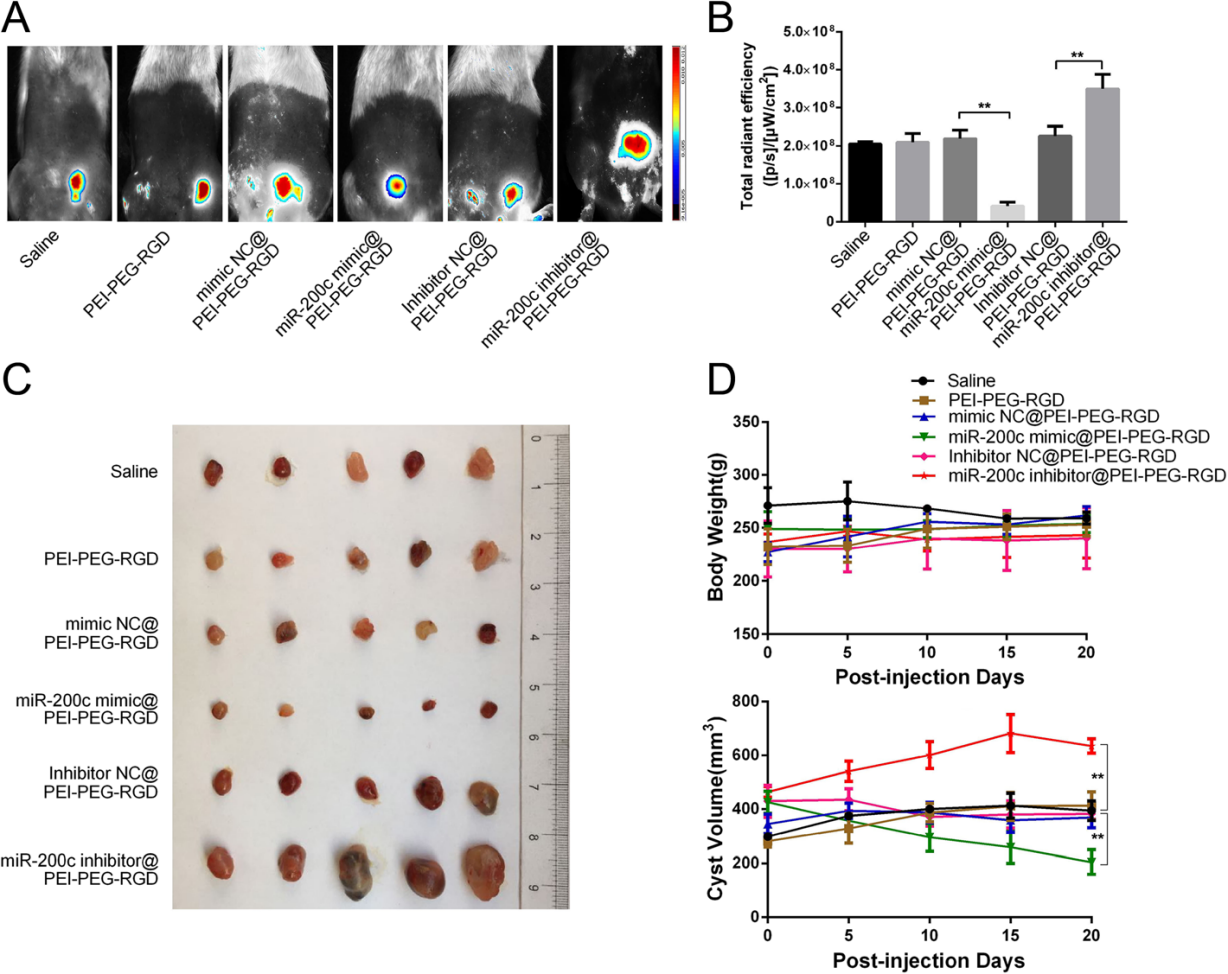
Therapeutic delivery of miR-200c reduced ectopic endometrial cysts in a rat model. **a** Near-infrared fluorescence imaging of ectopic endometrial cysts in a rat endometriosis model after injection of polyplexes. **b** Total fluorescence intensity in each group treated as in (**a**) (*n* = 5, **P < 0.01, Students *t* test). **c** Image of an ectopic endometrial cyst resected 20 days after injection of various polyplexes into rats. **d** Upper panel: body weights of each group measured every 5 days. Lower panel: volume of ectopic endometrial cysts measured during the 20 days of treatment (*n* = 5, **P < 0.01, Students *t* test, compared with the respective control group). PEI–PEG–RGD polyethylenimine–polyethylene glycol–arginine–glycine–aspartic acid, Mimic NC negative control miRNA mimic, Inhibitor NC negative control miRNA inhibitor

To investigate whether miR-200c is crucial for the progression of endometriosis in vivo, we established a rat endometriosis model to determine the effect of upregulation and downregulation of miR-200c. Near-infrared imaging showed that the intensity of fluorescence was significantly reduced in the miR-200c mimic treatment group compared with that in the mimic NC treatment group, while the intensity of fluorescence was significantly increased in the miR-200c inhibitor treatment group compared with that in the inhibitor NC treatment group (Fig. 5a, b). However, there was no significant difference in fluorescence intensity among the groups treated with saline, polyethylenimine–polyethylene glycol–arginine–glycine–aspartic acid (PEI–PEG–RGD), the mimic NC, or the inhibitor NC (Fig. 5a, b). When the rats were sacrificed 20 days after injection, we observed a significant reduction in the ectopic endometrial cyst volume in the miR-200c mimic treatment group compared with that in the mimic NC treatment group; in contrast, the volume of ectopic endometrial cysts was significantly increased in the miR-200c inhibitor treatment group compared with that in the inhibitor NC treatment group (Fig. 5c, d). Additionally, there were no significant differences among the treatment groups regarding body weight at the end of the experiment, and all of the rats survived (Fig. 5d). Therefore, miR-200c exerts a therapeutic effect in a rat model of endometriosis.

## Discussion

In recent years, increasing evidence has indicated that ncRNAs play a crucial regulatory role in the pathogenesis of many human diseases. miRNAs have been identified in relation to various biological processes and in the development and progression of diseases [21]. In the present study, we found that miR-200c was significantly downregulated in EC compared with in EN tissues. In addition, functional studies showed that overexpression of miR-200c inhibited the proliferation of HESCs, while the inhibition of miR-200c promoted cellular proliferation. Similar results were obtained from the migration assay. Furthermore, miR-200c mimic treatment reduced the volume of ectopic endometrial cysts, whereas miR-200c inhibitor treatment increased the volume of ectopic endometrial cysts in a rat endometriosis model.

Recently, lncRNAs, a class of transcripts longer than 200 nucleotides, have been shown to function as ceRNAs to inhibit miRNAs and thus regulate mRNA expression at the post-transcriptional level [19]. Accumulating evidence indicates that lncRNAs are involved in the regulation of tumor cell behavior, including proliferation, migration, and invasion [6, 10]. In endometriosis, the upregulation of H19 expression was shown to promote endometriotic stromal cell proliferation through the downregulation of let-7 to target IGF1R [12]. In our study, we used a miRNA–lncRNA interaction analysis program and identified lncRNA MALAT1 and XIST as possible targets of miR-200c. However, when we used qRT-PCR to determine expression of MALAT1 and XIST in the EC and EN tissues, we found that only MALAT1 was significantly upregulated in the EC tissues. In addition, miR-200c and MALAT1 levels were significantly negatively correlated. Further, we conducted luciferase reporter assays and transfected cells with the miR-200c mimic or inhibitor to demonstrate that MALAT1 is a target of miR-200c in HESCs.

Mounting evidence indicates that MALAT1 is one of the most abundant and well-conserved lncRNAs and is overexpressed in many human malignancies, including cancers of the nasopharynx, breast, prostate, bladder, and endometrium [22–26]. Although MALAT1 has been investigated in various human diseases, it has not been examined in endometriosis. In this study, we demonstrated that expression of MALAT1 is significantly increased during endometriosis. Functional studies showed that MALAT1 knockdown inhibits the proliferation and migration of HESCs, while downregulation of miR-200c expression restores these abilities in HESCs. These results suggested that MALAT1 regulated the proliferation and migration of HESCs in a miR-200c-dependent manner.

These findings prompted us to look further into the role of miR-200c in endometriosis. A study has shown that E-cadherin-negative cells are frequently observed in endometriotic tissues, whereas expression of mesenchymal markers (N-cadherin, Twist, and Snail) is upregulated in endometriosis relative to that in the healthy endometrium. The study suggests that EMT may be involved in the establishment of endometriotic lesions [27]. The hallmark of EMT is the replacement of E-cadherin by N-cadherin, which results in the loss of epithelial polarity and the gain of mesenchymal markers and motility [14]. miR-200c has also been shown to be involved in the EMT through targeting ZEB1 and ZEB2, the transcriptional repressors of E-cadherin [15]. Thus, we investigated whether miR-200c has the same functional roles in endometriosis. Indeed, the overexpression of miR-200c increased the level of E-cadherin and decreased the levels of ZEB1, ZEB2, and N-cadherin proteins, whereas an opposite effect was observed when miR-200c was suppressed using the miR-200c inhibitor in HESCs. Similarly, MALAT1-silencing led to the upregulation of E-cadherin and the downregulation of ZEB1, ZEB2, and N-cadherin in HESCs; however, the cotransfection of these cells with the miR-200c inhibitor abolished E-cadherin upregulation and ZEB1, ZEB2, and N-cadherin downregulation. Therefore, miR-200c suppresses EMT by targeting MALAT1 in endometriosis.

Of note, the miR200/MALAT1 sponge mediating the EMT processes in endometriosis is complex and never exclusive. Several studies found that during the transient window of mice embryo implantation, lysophosphatidic acid receptor 3 (LAPR3) is expressed exclusively in the endometrial epithelium of lumen and is regulated by progesterone and estrogen [28–30]. These findings indicate that LPAR3 may also have function in human reproduction. Wei et al. [31] have reported that LPAR3 expression was significantly reduced in the middle and later secretory endometrium of patients with endometriosis. In addition, LPAR3 signaling is critically involved in triggering β-catenin in stem and early progenitors [32]. Notably, endometrial stromal β-catenin is required for steroid-dependent mesenchymal–epithelial crosstalk and decidualization [33]; whether the LPAR3/β-catenin pathway also contributes to the EMT processes in endometriosis and its possible crosstalk with the miR200/MALAT1 sponge requires further research.

## Conclusions

We identified miR-200c as an EMT-suppressive miRNA in endometriosis that acts, at least in part, by downregulating the RNA level of MALAT1, which in turn functions as a ceRNA to upregulate ZEB1 and ZEB2 by sequestering miR-200c. The MALAT1/miR-200c sponge significantly affects the proliferation and migration of endometriotic stromal cells, indicating that this sponge may be a novel target for the diagnosis and treatment of endometriosis.

## Additional file

Additional file 1: Evaluation of cytotoxicity and transfection efficiency. (DOCX 14 kb)

## Abbreviations

ceRNA: Competing endogenous RNA
EdU: 5-Ethynyl-2′-deoxyuridine
EMT: Epithelial-to-mesenchymal transition
HESC: Primary endometrial stromal cell
LAPR3: Lysophosphatidic acid receptor 3
lncRNA: Long noncoding RNA
MALAT1: Metastasis-associated lung adenocarcinoma transcript 1
miRNA: MicroRNA
ncRNA: Noncoding RNA
PEI–PEG–RGD: Polyethylenimine–polyethylene glycol–arginine–glycine–aspartic acid
