# Supplementary material for: miR-200c suppresses endometriosis by targeting MALAT1 in vitro and in vivo
Source: Stem Cell Res Ther. 2017 Nov 7;8:251. doi: 10.1186/s13287-017-0706-z (PMC5678601; doi:10.1186/s13287-017-0706-z)
Supplement: Additional file 1: — Evaluation of cytotoxicity and transfection efficiency. (DOCX 14 kb) [file 13287_2017_706_MOESM1_ESM.docx]

**Supplementary Materials and Methods**

**Evaluation of transfection efficiency**

Transfection efﬁciency was evaluated by measuring the relative expression of miR-200c in HESCs. miR-200c loaded in PEI-PEG-RGD polymers (using the optimized N/P ratio) or Lipofectamine 2000 were transfected into cells in parallel wells. After incubation for 6 h, the medium was replaced, and the cells were cultured for an additional 24 h. Then, the cells were harvested, and the miRNA contents were assessed using qRT-PCR.

**Evaluation of cytotoxicity**

Twenty-four hours post-transfection, the cytotoxic effect of the polyplexes was assessed using Cell Counting Kit 8 (Dojindo, Tokyo, Japan) according to the manufacturer’s protocol. Briefly, 1×10^4^ cells/well were seeded in triplicate in 96-well culture plates, and the subsequent experiments were conducted when the cells reached 80% confluence. After removal of the culture medium, fresh medium containing 10% FBS and various concentrations (20-140 μg/ml) of miRNA NC@PEI-PEG-RGD nanoparticles (already optimized) were added to the cells. A negative control group was treated with an equivalent volume of PBS mixed with culture medium.
